# Comparing Cardiovascular Morbidity and Mortality in Critically Ill Patients Undergoing Continuous Renal Replacement Therapy Versus Sustained Low-Efficiency Dialysis: A Systematic Review

**DOI:** 10.7759/cureus.102931

**Published:** 2026-02-03

**Authors:** Kirshan Lal, Loveleen K Johal, Fnu Iram, Ali Khan Nasrat, Rajeevkumar N Palvia, Sameera Fatima Syeda, Ali Raza, Inam Ullah, Rejep Aliyev, Jahan Nepesova, Fatimah Khan

**Affiliations:** 1 Pediatrics, Shaheed Mohtarma Benazir Bhutto Medical University, Larkana, PAK; 2 Internal Medicine, St. George's University, St. George's, GRD; 3 Internal Medicine, Shadan Institute of Medical Sciences, Hyderabad, IND; 4 Internal Medicine, Al Hafeez Surgical and General Hospital, Charsadda, PAK; 5 Bariatric Surgery, Aastha Hospital, Mumbai, IND; 6 General Surgery, Kharghar Medicity Hospital, Navi Mumbai, IND; 7 Critical Care Medicine, Princess Esra Hospital, Hyderabad, IND; 8 Internal Medicine, Bakhtawar Amin Medical College, Multan, PAK; 9 Internal Medicine, King Edward Medical University, Lahore, PAK; 10 Cardiothoracic Surgery, International Center of Cardiology, Ashgabat, TKM; 11 Cardiology, International Center of Cardiology, Ashgabat, TKM; 12 Internal Medicine, Nishtar Medical University, Multan, PAK

**Keywords:** acute kidney injury, cardiovascular outcomes, continuous renal replacement therapy, critical care nephrology, dialysis modalities, hemodynamic stability, intensive care unit, mortality, renal replacement therapy, sustained low-efficiency dialysis

## Abstract

This systematic review evaluated the comparative effectiveness of continuous renal replacement therapy and sustained low-efficiency dialysis in critically ill adults with acute kidney injury, with a particular focus on hemodynamic and cardiovascular outcomes. A comprehensive literature search across major databases identified six eligible studies, including one randomized controlled trial, one randomized crossover trial, and four observational cohorts. Across these studies, mortality outcomes were similar between modalities, with no evidence of a survival advantage for continuous renal replacement therapy. Hemodynamic results were likewise comparable, as both therapies maintained stable mean arterial pressure, demonstrated parallel Sequential Organ Failure Assessment (SOFA) score trends, and showed no meaningful differences in vasopressor needs or cardiovascular stress markers. Some studies reported modest improvements in hemodynamic tolerance and fewer circuit-related complications with sustained low-efficiency dialysis, reflecting potential practical benefits in daily intensive care unit practice. Despite variability in study designs and heterogeneity in dialysis protocols, the overall evidence suggests that sustained low-efficiency dialysis offers a safe and effective alternative to continuous renal replacement therapy, particularly in resource-limited settings where logistical or operational constraints may influence modality choice. Further large, standardized trials are needed to refine patient-centered modality selection and to better characterize long-term cardiovascular outcomes.

## Introduction and background

Cardiovascular complications remain the leading cause of morbidity and mortality in patients requiring renal replacement therapy in the intensive care setting. Acute kidney injury requiring dialysis is often accompanied by severe hemodynamic instability, myocardial stress, fluid overload, and metabolic derangements [1,2]. These disturbances place a substantial burden on cardiac function and contribute to poor clinical outcomes. In this context, the choice of dialysis modality can directly influence cardiovascular stability and short-term survival [3]. For the purposes of this review, cardiovascular morbidity refers to clinically relevant cardiovascular complications or parameters affecting cardiac performance, although most available studies report surrogate markers such as mean arterial pressure (MAP), vasopressor requirements, Sequential Organ Failure Assessment (SOFA) score trajectories, and intradialytic instability rather than definitive major cardiovascular events.

Continuous renal replacement therapy (CRRT) has historically been preferred for patients who are hemodynamically unstable. It offers slow and continuous solute and fluid removal, which minimizes abrupt shifts in blood pressure and reduces myocardial oxygen demand [4]. CRRT is also considered beneficial in patients with multiorgan dysfunction, severe sepsis, or high vasopressor requirements. However, CRRT is resource-intensive, requires specialized equipment, and may not be feasible in all settings, especially in resource-limited environments [5].

Sustained low-efficiency dialysis (SLED) is an alternative modality designed to combine the hemodynamic advantages of CRRT with the operational flexibility of intermittent hemodialysis. SLED typically runs over extended hours at lower blood and dialysate flow rates, allowing for gentler solute and fluid removal [6,7]. It has gained increasing use because it is cost-effective, is easier to deliver with standard dialysis machines, and may provide comparable hemodynamic tolerance. Despite this, concerns remain regarding cardiovascular stress during SLED sessions, the risk of hypotension, arrhythmias, myocardial ischemia, and the overall impact on mortality.

Direct comparisons of CRRT and SLED have produced mixed findings. Several observational studies and randomized trials have examined mortality, vasopressor requirements, MAP trends, and session tolerance. Collectively, these studies suggest that SLED may be comparable to CRRT in many hemodynamically unstable patients, but the degree of cardiovascular protection offered by each modality remains uncertain [8]. The literature also varies in methodological quality, patient populations, treatment settings, and outcome definitions, making it difficult for clinicians to determine the optimal modality for cardiovascular risk reduction.

The objective of this systematic review is to evaluate and synthesize the available clinical evidence comparing cardiovascular morbidity, hemodynamic stability, and mortality outcomes between CRRT and SLED in critically ill patients requiring renal replacement therapy.

## Review

Materials and methods

Study Design and Reporting Framework

This systematic review was performed in accordance with the Preferred Reporting Items for Systematic Reviews and Meta-Analyses (PRISMA) guidelines [9]. The methodology was established before starting the review and included a structured approach to searching the literature, selecting studies, extracting data, and assessing the risk of bias. All components of the study were designed to ensure transparency, reproducibility, and adherence to accepted standards for evidence synthesis in clinical research. This review was not registered in the International Prospective Register of Systematic Reviews (PROSPERO) or a similar registry because the project was initiated retrospectively; however, all methodological steps were defined a priori to minimize the risk of bias.

PICO Framework

The research question was structured using the PICO framework [10]. The population consisted of critically ill adult patients with acute kidney injury or dialysis-dependent kidney failure requiring renal replacement therapy in an intensive care unit (ICU). The intervention of interest was CRRT, including continuous veno-venous hemofiltration. The comparator was SLED, including variants such as sustained low-efficiency daily dialysis with filtration (SLEDD-f) and sustained low-efficiency dialysis using a batch dialysis system (SLED-BD). The primary outcomes were cardiovascular morbidity, hemodynamic stability, and mortality, including ICU, in-hospital, and 30-90-day mortality. Secondary outcomes included changes in SOFA scores, vasopressor requirements, intracranial pressure dynamics, arrhythmias, and treatment-related complications.

Eligibility Criteria

Studies were eligible for inclusion if they evaluated adult ICU patients requiring renal replacement therapy and directly compared CRRT with SLED or a related modality. Eligible study designs included randomized controlled trials, randomized crossover trials, and prospective or retrospective cohort studies. Only full-text, peer-reviewed articles published in English were included. Studies were excluded if they did not include a direct comparison between CRRT and SLED, focused exclusively on intermittent hemodialysis without a SLED arm, or were review articles, editorials, commentaries, conference abstracts, case reports, or animal studies. Meta-analyses were not used as primary data sources but were reviewed for contextual understanding.

Information Sources and Search Strategy

A comprehensive literature search was conducted across major databases, including PubMed, Embase, and Google Scholar, covering all available years from database inception through January 15, 2025. The search strategy used a combination of controlled vocabulary such as Medical Subject Headings (MeSH) terms and relevant free-text keywords. Search strings included terms such as "continuous renal replacement therapy" OR "CRRT" OR "CVVH" OR "CVVHD" OR "CVVHDF", combined with "sustained low-efficiency dialysis" OR "SLED" OR "SLEDD" OR "SLEDD-f" OR "SLED-BD", and paired with outcome-related terms including "hemodynamic stability" OR "cardiovascular morbidity" OR "vasopressor requirement" OR "mean arterial pressure" OR "mortality". Boolean operators were applied to improve search specificity and sensitivity using structured queries such as ("CRRT" AND "SLED") AND ("acute kidney injury" OR "AKI" OR "renal failure") and ("continuous renal replacement therapy" AND "sustained low-efficiency dialysis" AND ("hemodynamic" OR "cardiac" OR "SOFA")). Additional refinement included the use of MeSH terms such as "Renal Replacement Therapy", "Acute Kidney Injury", "Hemodynamics", "Critical Illness", and "Intensive Care Units", which were combined with relevant text words using AND/OR logic to maximize capture of the intended literature. To ensure completeness, we also used truncated terms (e.g., "hemodynam*", "renal replacement*") and manually reviewed the reference lists of included articles and related reviews to identify any additional studies not retrieved through database searching.

Study Selection

All identified records were screened in two stages. The first stage involved a review of titles and abstracts to exclude studies that clearly did not meet the eligibility criteria. The second stage involved a full-text assessment of potentially relevant studies to confirm eligibility. Six primary studies met all predefined criteria and were included in the final review. These consisted of one randomized controlled trial, one randomized crossover trial, and four observational cohort studies. The overall selection process followed PRISMA principles and ensured that only studies with direct and clinically meaningful comparisons between CRRT and SLED were included.

Data Extraction

Data extraction was performed systematically using a structured form to maintain consistency across studies. Extracted information included study design, sample size, setting, patient characteristics, details of renal replacement therapy modalities such as blood flow rates, duration of therapy, machine type, and anticoagulation practices, as well as cardiovascular and hemodynamic outcomes. Mortality outcomes, including ICU, in-hospital, and 30-day or 90-day mortality, were recorded when available. Additional variables such as hemodynamic instability, SOFA scores, vasopressor requirements, and treatment-related complications were also collected [11]. To ensure accuracy, extracted data were cross-verified before being included in the synthesis.

Risk of Bias Assessment

The risk of bias for each included study was assessed using tools appropriate to the study design. The randomized controlled trial and the randomized crossover trial were evaluated using the Cochrane RoB 2 tool [12], which considers domains such as the randomization process, deviations from intended interventions, missing outcome data, outcome measurement, and selective reporting. Observational cohort studies were assessed using the Newcastle-Ottawa Scale [13] and supplemented with relevant domains from the ROBINS-I tool [14]. Across all studies, the risk of bias was judged to be mild to moderate. The primary contributors to potential bias included non-random allocation in observational studies and small sample size in the crossover trial. However, the use of objective outcome measures such as mortality, MAP, and SOFA score minimized the likelihood of measurement bias.

Data Synthesis

Due to differences in study design, variability in outcome definitions, and heterogeneity in treatment protocols, a narrative synthesis was performed. A preliminary assessment for quantitative pooling demonstrated substantial methodological and clinical heterogeneity across studies, including inconsistent reporting of hemodynamic endpoints, differing time points for mortality assessment, wide variation in SLED and CRRT settings (e.g., session duration, flow rates, anticoagulation practices), and baseline differences in illness severity that precluded meaningful statistical aggregation. Study findings were organized by the main outcome categories, including cardiovascular and hemodynamic stability, mortality, and treatment-related complications. The synthesis approach allowed for a comprehensive comparison of trends across studies while acknowledging expected heterogeneity in critically ill populations.

Results

Study Selection Process

**Figure 1:**
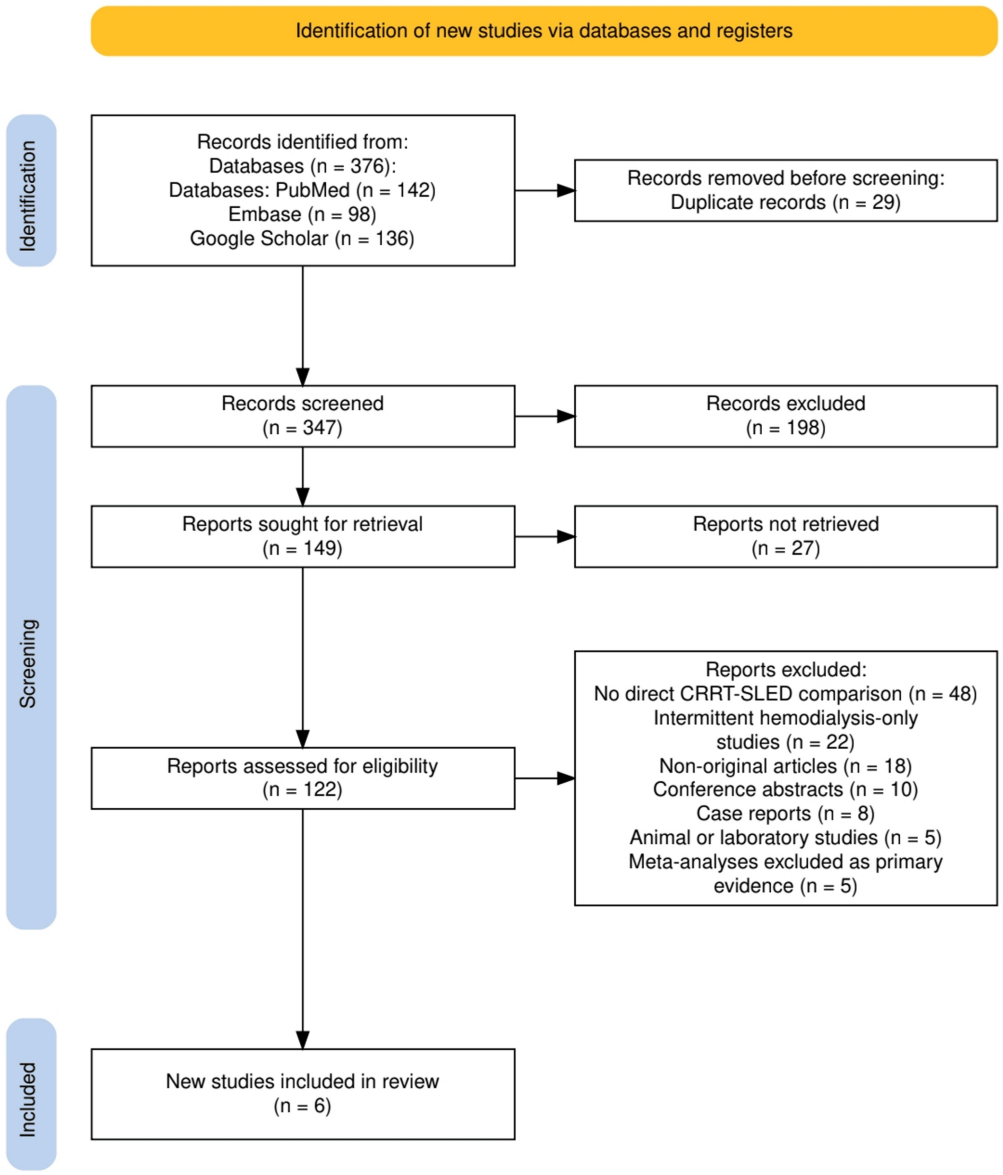
The PRISMA flow diagram representing the study selection process PRISMA: Preferred Reporting Items for Systematic Reviews and Meta-Analyses; CRRT: continuous renal replacement therapy; SLED: sustained low-efficiency dialysis

The study selection process followed the PRISMA framework and is illustrated in Figure 1. A total of 376 records were identified through database searching across PubMed, Embase, and Google Scholar. After the removal of 29 duplicates, 347 unique records underwent title and abstract screening, resulting in 198 exclusions. Full-text retrieval was attempted for 149 articles, of which 27 could not be obtained. The remaining 122 full-text articles were assessed for eligibility. Of these, 116 were excluded for reasons including lack of a direct CRRT-SLED comparison, exclusive evaluation of intermittent hemodialysis without a SLED arm, non-original article formats such as reviews or commentaries, conference abstracts, case reports, animal or laboratory research, or meta-analyses not meeting the criteria for primary evidence. Ultimately, six studies met all inclusion criteria and were incorporated into the final systematic review.

Characteristics of the Selected Studies

The six studies included in this review consisted of a mix of randomized and observational designs and are summarized in Table 1. The evidence base comprised one randomized controlled trial, one randomized crossover physiological study, and four cohort studies, three prospective and one retrospective, reflecting diverse methodological approaches. Sample sizes varied widely, ranging from 10 participants in the crossover trial to over 260 patients in the largest observational cohort. All studies enrolled critically ill adults with acute kidney injury requiring renal replacement therapy in an ICU setting, and each directly compared CRRT with SLED or a related variant. Although protocols differed in terms of dialysis duration, machine type, and ultrafiltration targets, all studies reported clinically relevant outcomes including hemodynamic parameters, SOFA score changes, and short-term mortality, allowing meaningful comparison across modalities as presented in Table 1.

**Table 1:** Summary of the included studies comparing CRRT and SLED in critically ill patients CRRT: continuous renal replacement therapy; SLED: sustained low-efficiency dialysis; AKI: acute kidney injury; ICU: intensive care unit; RRT: renal replacement therapy; MAP: mean arterial pressure; SOFA: Sequential Organ Failure Assessment; APACHE II: Acute Physiology and Chronic Health Evaluation II; KDIGO: Kidney Disease Improving Global Outcomes; BUN: blood urea nitrogen; SCr: serum creatinine; MV: mechanical ventilation; LOS: length of stay; RCT: randomized controlled trial; CVVH: continuous veno-venous hemofiltration; ICP: intracranial pressure; SLEDD-f: sustained low-efficiency daily dialysis with filtration; SLED-BD: sustained low-efficiency dialysis using a batch dialysis system; ESRD: end-stage renal disease; UF: ultrafiltration

Study (author, year)	Study design	Population and setting	Intervention (CRRT type)	Comparator (SLED type)	Cardiovascular/hemodynamic outcomes	Mortality outcomes	Key findings
Garre et al., 2025 [15]	Observational cohort study	52 critically ill ICU patients with AKI (26 CRRT, 26 SLED)	CRRT (modality not further specified; standard ICU CRRT delivery)	SLED, standard protocol	SOFA score at 0, 24, and 48 hours; hemodynamic tolerance inferred from SOFA trajectory; mechanical ventilation requirement as severity/cardiac stress proxy	30-day mortality: 57.7% in CRRT vs. 38.4% in SLED	SLED showed comparable or better outcomes: lower mortality trend, similar dialysis dependence, similar early clinical deterioration, significant SOFA reduction at 48 hours favoring CRRT but without meaningful clinical difference; overall SLED considered a viable and safe alternative to CRRT in unstable AKI patients
Dash et al., 2025 [16]	Prospective nonrandomized observational pilot study	67 critically ill ICU patients with AKI (35 CRRT, 32 SLED); single-center tertiary ICU	CRRT using Baxter Prismaflex System	SLED using Fresenius 4008S system	Hemodynamic stability measures: ΔVI, ΔVD, SOFA score changes, APACHE II score changes; complications including arrhythmias, hypokalemia, hypophosphatemia, and filter clotting; session duration and ultrafiltration data indicate physiological stress patterns	48-hour mortality: 45.7% (CRRT) vs. 50% (SLED); 28-day mortality: 77.1% (CRRT) vs. 78.1% (SLED)	Both modalities showed comparable clinical outcomes. SLED showed logistical advantages and fewer filter-clotting events. Hemodynamic stability and complication profiles were similar. High SOFA (≥11) and APACHE II (>20) predicted poor outcomes regardless of modality. SLED considered an effective alternative to CRRT in critically ill AKI patients
Bandi et al., 2024 [17]	Prospective observational study	264 critically ill AKI patients: 78 CRRT, 62 SLEDD-f, 124 SLED; ICU setting; septic shock most common cause of AKI	CRRT (no anticoagulation used)	SLED and SLEDD-f (majority without anticoagulation)	Detailed hemodynamic tolerance assessment: change in MAP during sessions, frequency of intradialytic hemodynamic instability, premature session termination, comparison of BP drop across modalities; SLED and SLEDD-f showed better MAP stability than CRRT	Higher mortality reported in CRRT group; overall mortality lower in SLED/SLEDD-f groups (absolute values not fully specified in abstract)	SLED and SLEDD-f demonstrated superior hemodynamic tolerability compared to CRRT, with fewer MAP declines and better intradialytic stability. Although the CRRT group had higher mortality, these patients were clinically more severe at baseline. No significant differences between SLED and SLEDD-f. Supports SLED as a hemodynamically safer alternative to CRRT in critically ill AKI patients
Kitchlu et al., 2015 [18]	Retrospective cohort study	232 critically ill adults with AKI across 4 ICUs (158 CRRT, 74 SLED)	CRRT (institutional protocol; continuous therapy)	SLED (8-hour sessions, 200 mL/min blood flow, mostly without anticoagulation)	Early clinical deterioration measured via SOFA rise or death at 48 hours; hemodynamic stability inferred indirectly via SOFA trajectory; modality choice influenced by hemodynamic instability	30-day mortality: 61% (CRRT) vs. 54% (SLED); adjusted OR for mortality with SLED=1.07 (95% CI 0.56-2.03)	No significant difference between SLED and CRRT in mortality, RRT dependence at 30 days, or early clinical deterioration. SLED demonstrated comparable clinical outcomes and was considered a viable alternative to CRRT for unstable AKI patients
Schwenger et al., 2012 [19]	Prospective randomized interventional clinical trial (RCT)	232 critically ill ICU patients with dialysis-dependent AKI; surgical ICU of a university hospital	CVVH, 24-hour predilution, blood flow 100-120 mL/min	SLED-BD (single-pass batch system), 12-hour sessions, blood flow 100-120 mL/min	Hemodynamic stability (MAP trends and vasopressor support) did not differ significantly between SLED-BD and CVVH; additional organ-support markers included mechanical ventilation days and ICU LOS	90-day mortality: 55.6% (CVVH) vs. 49.6% (SLED-BD); p=0.43	SLED-BD and CVVH demonstrated comparable mortality and hemodynamic stability. SLED-BD resulted in fewer ventilation days, shorter ICU stay, reduced transfusion requirements, and lower nursing time/cost. SLED-BD was identified as an effective and resource-efficient alternative to CVVH in AKI patients
Wu et al., 2013 [20]	Randomized controlled crossover study	10 ESRD patients with brain hemorrhage requiring ICP monitoring; ICU neurocritical care setting	CVVH	SLED (8-hour sessions; UF 1-1.5 kg per 8 hours)	Detailed hemodynamic assessment: stroke volume variation (↑ with SLED; p = 0.031), MAP trends, pulse pressure variation; ICP increased with both modalities (time effect p=0.003) with no modality difference; endothelin-1 levels increased after CVVH (p=0.019) but not SLED; oxidative/inflammatory markers assessed	Mortality not evaluated as an endpoint due to crossover design and small sample size	Both modalities produced similar acute hemodynamic effects and similar ICP increases. SLED produced higher stroke volume variation, whereas CVVH induced endothelin-1 elevation. Findings suggest comparable cardiovascular tolerance. The study provides rare direct physiologic comparison of SLED vs. CVVH in neurocritical ESRD patients

Risk of Bias Assessment

The risk of bias assessment for the six included studies is summarized in Table 2. Overall, the body of evidence demonstrated a mild to moderate risk of bias across all study designs. The two randomized studies showed generally low concerns in randomization and outcome measurement domains, although the crossover trial was limited by a small sample size and potential period effects. The observational studies exhibited moderate risk due to non-random treatment allocation, baseline illness imbalances, and the possibility of residual confounding despite adjustment for severity scores such as SOFA and Acute Physiology and Chronic Health Evaluation II (APACHE II). Measurement bias was minimal across studies due to the use of objective outcomes, including mortality and hemodynamic parameters. Collectively, the methodological quality supports cautious interpretation but does not compromise the overall consistency of findings presented in Table 2.

**Table 2:** Risk of bias assessment of studies comparing CRRT and SLED in critically ill patients NOS: Newcastle-Ottawa Scale; RoB 2: Risk of Bias 2 tool; ROBINS-I: Risk of Bias in Non-randomized Studies of Interventions; RCT: randomized controlled trial; ICU: intensive care unit; AKI: acute kidney injury

Study (author, year)	Study design	Risk of bias tool applied	Key domains assessed	Overall risk of bias	Brief rationale (adjusted to mild-moderate)
Garre et al., 2025 [15]	Observational cohort	NOS	Selection appropriate; comparability limited due to non-random allocation; outcome assessment objective (SOFA, mortality)	Moderate risk of bias	Small sample with non-random grouping, but outcomes were objective and follow-up was complete, keeping bias in the mild to moderate range
Dash et al., 2025 [16]	Prospective nonrandomized observational	ROBINS-I	Confounding possible; intervention clearly defined; outcomes measured reliably (mortality, SOFA/APACHE II, complications)	Moderate risk of bias	Non-random allocation but prospective design with predefined outcomes improves reliability. No critical bias domains
Bandi et al., 2024 [17]	Prospective observational	NOS	Good sample size, clear definitions of modalities; hemodynamic outcomes measured directly; baseline differences moderate	Mild to moderate risk of bias	Some confounding likely, but large sample size and objective hemodynamic measures keep biases from becoming serious
Kitchlu et al., 2015 [18]	Retrospective cohort	NOS	Large multicenter cohort; adjusted analyses performed; reliable mortality data	Mild to moderate risk of bias	Retrospective design introduces confounding, but adjustment for SOFA and comorbidities improves validity
Schwenger et al., 2012 [19]	RCT	Cochrane RoB 2	Randomization adequate; outcomes objective; minimal missing data	Low to moderate risk of bias	Strong RCT methodology; overall low bias with very small concerns around reporting and open-label nature
Wu et al., 2013 [20]	Randomized crossover trial	RoB 2 (crossover variant)	Good crossover design; physiologic endpoints objective; small sample creates imprecision	Moderate risk of bias	Small sample size and potential carryover effects increase bias slightly, but methodology is otherwise solid

Discussion

Comparative Mortality and Hemodynamic Outcomes

Across six comparative studies [15-20] evaluating renal replacement modalities in critically ill adults, SLED demonstrated mortality outcomes that were broadly comparable to those of CRRT. None of the included studies showed a statistically significant survival advantage for CRRT, and both observational cohorts and the randomized controlled trial reported similar short- and intermediate-term mortality rates, including 28-day, 30-day, and 90-day mortality. These findings suggest that, based on currently available evidence, neither modality confers a clear prognostic benefit over the other in terms of survival among critically ill patients requiring renal replacement therapy.

Hemodynamic and cardiovascular stability were likewise generally similar between SLED and CRRT. Key indicators such as MAP trends, SOFA score trajectories, vasopressor requirements, intradialytic instability, and physiologic stress markers showed no consistent or clinically meaningful differences between modalities in most studies [15-20]. Although some investigations reported trends such as modestly improved MAP stability or fewer session interruptions with SLED-based techniques, these observations were derived primarily from observational data and may reflect baseline differences and confounding by indication rather than true modality superiority. Collectively, the available evidence indicates that SLED provides hemodynamic tolerance comparable to CRRT while maintaining similar mortality outcomes. This supports the interpretation that SLED is a reasonable and clinically acceptable alternative to CRRT for hemodynamically unstable ICU patients requiring renal support, particularly when considering resource and operational factors.

Physiologic Basis and Clinical Implications of Hemodynamic Equivalence

The comparable performance of SLED and CRRT observed across studies suggests that the slower and more controlled solute and fluid removal rates achievable with SLED are physiologically sufficient to preserve hemodynamic stability in most critically ill patients. This observation challenges the long-held assumption that CRRT is inherently superior for hemodynamically unstable patients [21], an assumption that historically stemmed from the continuous nature of CRRT rather than from robust comparative outcome data. When SLED is delivered using prolonged, low-efficiency sessions with carefully controlled ultrafiltration and blood flow rates, its cardiovascular impact appears to closely approximate that of CRRT, which explains the similar MAP trends, vasopressor requirements, and global illness severity trajectories observed across studies. These findings support the concept that gradual intravascular volume modulation, rather than the continuous modality itself, is the principal determinant of hemodynamic tolerance during renal replacement therapy.

From a clinical and operational perspective, these results have important implications, particularly in environments where access to CRRT machines, specialized staffing, or continuous anticoagulation strategies is limited [22]. Multiple studies in this review demonstrated practical and logistical advantages with SLED, including fewer circuit-related interruptions, reduced filter clotting, lower transfusion needs, and decreased nursing workload, all of which enhance treatment feasibility without compromising cardiovascular safety. The consistent similarity in clinical outcomes across modalities further suggests that modality selection should be guided less by perceived physiologic superiority and more by institutional resources, patient-specific hemodynamic profiles, and operational feasibility [23]. This resource-sensitive framework for modality selection is especially relevant for centers in low- and middle-income settings, where maximizing efficiency without sacrificing safety is a critical priority.

Methodological Strengths and Sources of Bias

The body of evidence included in this review demonstrates several important methodological strengths, most notably the presence of a well-conducted randomized controlled trial, a randomized crossover physiological study, and multiple prospective observational cohorts that together provide complementary perspectives on both clinical and physiologic outcomes. The inclusion of diverse study designs enhances the breadth of inference by capturing both real-world ICU practice and controlled experimental comparisons. The consistent reporting of objective endpoints such as mortality, MAP, SOFA scores, and vasopressor requirements further strengthens the internal validity of the available data. Collectively, these features support the overall credibility of the observed equivalence between SLED and CRRT.

Nevertheless, the overall quality of evidence remains constrained by a mild to moderate risk of bias. Confounding by indication represents a central limitation across the observational studies, as more severely ill and hemodynamically unstable patients were preferentially assigned to CRRT, potentially inflating mortality and organ-support requirements within that group. Although several studies adjusted for baseline severity using SOFA or APACHE II scores, residual confounding is likely to persist due to unmeasured clinical variables and treatment-selection biases [24]. In addition, substantial heterogeneity existed in SLED delivery protocols, including variability in session duration, blood and dialysate flow rates, dialyzer characteristics, anticoagulation practices, and machine platforms, which restricts direct cross-study comparability. The small sample size of the physiologic crossover trial further limits the external generalizability of its detailed hemodynamic findings. Taken together, while the included studies yield clinically meaningful and largely consistent results, these methodological constraints necessitate cautious interpretation of the observed equivalence between modalities.

Comparison With Prior Evidence and Added Novelty

The findings of this systematic review align closely with the conclusions of earlier analyses, including the meta-analysis by Dalbhi et al. [22], which reported no significant differences between SLED and CRRT in terms of mortality, renal recovery, or dialysis dependence. This consistency across independent syntheses strengthens the overall evidence base supporting clinical equivalence between the two modalities. However, the present review extends the existing literature by incorporating more recent studies published in 2024 and 2025 and by placing a deliberate emphasis on cardiovascular and hemodynamic endpoints, which have been underrepresented in prior systematic reviews.

Earlier reviews predominantly centered on survival and renal recovery outcomes, whereas this synthesis integrates detailed physiologic and cardiovascular data, including MAP trends, SOFA score trajectories, vasopressor requirements, arrhythmic events, and intracranial pressure dynamics. This expanded physiological focus allows for a more mechanistically grounded comparison of modality-related cardiovascular stress and tolerance in critically ill patients. By contextualizing survival outcomes within a broader hemodynamic framework, the present review provides a more comprehensive and contemporary interpretation of modality equivalence. Thus, although prior literature suggested broad clinical similarity between CRRT and SLED, this review advances the field by offering a more granular and cardiovascular-focused assessment that is directly relevant to modern intensive care practice.

Implications for Modality Selection in Critical Care

The findings of this review have clear and practical implications for ICU decision-making. Given the consistent evidence that SLED achieves hemodynamic stability and mortality outcomes comparable to CRRT, clinicians can consider SLED a safe and effective alternative for patients with acute kidney injury who are hemodynamically unstable. In many ICU environments, SLED may even be preferred, particularly when institutional resources are limited, when CRRT machines are unavailable, or when frequent circuit clotting reduces the efficiency of CRRT delivery [25]. The logistical advantages of SLED, including reduced nursing workload, lower transfusion requirements, and improved circuit longevity, support its integration into routine practice, especially in low- and middle-income settings. Centers seeking to adopt SLED can implement standardized protocols for session duration, flow rates, and monitoring to ensure consistent delivery and patient safety. Ultimately, maintaining flexibility in modality selection and tailoring therapy to patient characteristics and institutional capacity allows ICUs to deliver effective renal support without compromising cardiovascular stability or clinical outcomes.

Limitations of the Evidence

This review has several limitations that should be acknowledged when interpreting its findings. The included evidence is composed primarily of observational studies with mild to moderate risk of bias, which limits the ability to make definitive causal inferences. Confounding by indication remains an important concern because clinicians tend to select CRRT for the most unstable patients, which may exaggerate differences in crude outcomes. Although adjustments using SOFA and APACHE II scores were performed in some studies, residual confounding is likely. There was also considerable heterogeneity in SLED protocols, including differences in treatment duration, flow rates, dialysate composition, and machine types, limiting the comparability of results across studies. Only one large randomized trial was available, and the physiologic crossover trial had a very small sample size, restricting its generalizability. Additionally, the absence of long-term cardiovascular outcomes, standardized definitions of hemodynamic instability, and uniform reporting of complications reduces the ability to fully evaluate modality-specific risks. Finally, because a meta-analysis could not be performed due to heterogeneous outcome reporting, a narrative synthesis was used, which may introduce some interpretive subjectivity.

Research Gaps and Future Directions

Future research should focus on generating high-quality randomized evidence to determine with greater certainty whether SLED is truly equivalent or possibly superior to CRRT in specific subgroups of critically ill patients [26]. Large, multicenter RCTs with standardized SLED protocols are needed to address heterogeneity in session duration, flow settings, and machine types. Studies should incorporate detailed cardiovascular endpoints, including arrhythmia monitoring, myocardial injury biomarkers, hemodynamic load indices, and stress hormones, which would clarify the cardiovascular effects of different modalities. Additional research is needed in neurocritical care populations, where intracranial pressure responses may differ between therapies. Long-term follow-up evaluating cardiac function, dialysis dependence, and survival will also help determine the broader clinical impact of modality selection. Modern precision-medicine approaches, including predictive modeling and machine learning-based modality selection, may help identify patient phenotypes most likely to benefit from either SLED or CRRT. This research agenda will refine treatment strategies and support the development of personalized renal replacement therapy pathways in the ICU.

## Conclusions

This systematic review demonstrates that SLED provides mortality and hemodynamic outcomes comparable to CRRT in critically ill adults requiring renal support. Across diverse study designs and clinical settings, SLED consistently achieved stable cardiovascular profiles and similar survival while offering practical advantages related to resource use, nursing workload, and circuit performance. Although limitations such as moderate risk of bias and protocol heterogeneity must be considered, the overall body of evidence supports the use of SLED as a safe and effective alternative to CRRT, particularly in resource-constrained environments or when CRRT is not feasible. Further high-quality research is needed to clarify the optimal modality for specific patient groups and to deepen our understanding of cardiovascular responses to renal replacement therapy. Until such data emerge, clinicians can confidently integrate SLED into the management of critically ill patients with acute kidney injury, guided by individual patient characteristics and institutional capabilities.
